# Investigating the Interactive Effects of Sex Steroid Hormones and Brain-Derived Neurotrophic Factor during Adolescence on Hippocampal NMDA Receptor Expression

**DOI:** 10.1155/2018/7231915

**Published:** 2018-01-31

**Authors:** Cushla R. McCarthny, Xin Du, YeeWen Candace Wu, Rachel A. Hill

**Affiliations:** ^1^Department of Psychiatry, School of Clinical Sciences, Monash Medical Centre, Monash University, Clayton, VIC, Australia; ^2^The Florey Institute of Neuroscience and Mental Health, University of Melbourne, Parkville, VIC, Australia; ^3^Department of Psychiatry and Behavioral Sciences, Johns Hopkins University School of Medicine, Baltimore, MD, USA

## Abstract

Sex steroid hormones have neuroprotective properties which may be mediated by brain-derived neurotrophic factor (BDNF). This study sought to determine the interactive effects of preadolescent hormone manipulation and BDNF heterozygosity (^+/−^) on hippocampal NMDA-R expression. Wild-type and BDNF^+/−^ mice were gonadectomised, and females received either 17*β*-estradiol or progesterone treatment, while males received either testosterone or dihydrotestosterone (DHT) treatment. Dorsal (DHP) and ventral hippocampus (VHP) were dissected, and protein expression of GluN1, GluN2A, GluN2B, and PSD-95 was assessed by Western blot analysis. Significant genotype × OVX interactions were found for GluN1 and GluN2 expression within the DHP of female mice, suggesting modulation of select NMDA-R levels by female sex hormones is mediated by BDNF. Furthermore, within the DHP BDNF^+/−^ mice show a hypersensitive response to hormone treatment on GluN2 expression which may result from upstream alterations in TrkB phosphorylation. In contrast to the DHP, the VHP showed no effects of hormone manipulation but significant effects of genotype on NMDA-R expression. Castration had no effect on NMDA-R expression; however, androgen treatment had selective effects on GluN2B. These data show case distinct, interactive roles for sex steroid hormones and BDNF in the regulation of NMDA-R expression that are dependent on dorsal versus ventral hippocampal region.

## 1. Introduction

Sex steroid hormones have been widely investigated in the brain and are known to exert a range of neuroprotective effects including antiapoptotic and antioxidant properties in addition to the enhancement of neurogenesis and synaptic plasticity [1, 2]. The predominant female sex steroid hormone, 17*β*-estradiol, is particularly renowned for its neuroprotective properties, which it exerts via activation of its high-affinity receptor ERs *α* and *β* and the recently characterised G-protein-coupled receptor (GPER1). Estradiol's effect at the synapse may not be exclusively mediated via ER-dependent signalling; alternatively, estradiol may operate in a more indirect fashion, by activating growth factors such as BDNF [3]. BDNF plays an integral role in the growth and survival of new synapses in addition to the strengthening of existing connections [4]. Abnormal BDNF expression and activity have been associated with a range of neurological disorders. In particular, the Val66Met polymorphism, which leads to a functional reduction in activity-dependent release of BDNF, has been linked to psychiatric illnesses such as major depression and schizophrenia [5].

Converging evidence suggests that BDNF is regulated by estradiol levels and may have a role in mediating the synaptic changes observed in response to fluctuating estradiol levels [4]. Hippocampal BDNF mRNA expression levels have been shown to fluctuate in response to cycling estradiol throughout the estrus cycle [6]. Moreover, ovariectomy (OVX) causes a reduction in the levels of BDNF mRNA in the hippocampus and estradiol replacement has the ability to restore this reduction in rats [7]. In addition, our laboratory found that hippocampal expression of BDNF correlates with circulating levels of estradiol during adolescent development [8], and prepubescent ovariectomy reduced hippocampal BDNF, while treatment with estradiol but not progesterone restored BDNF expression [9].

The discovery of an estrogen response element- (ERE-) like motif in the BDNF gene promotor region provides a possible mechanism through which estradiol is able to directly regulate BDNF expression [10]. An alternative theory is that both estradiol and BDNF act synergistically, by converging at the same intracellular signalling cascade, thus producing the same observable changes at the synapse. For example, Briz and Baudry [11] found that the application of an extracellular signal-regulated kinase (ERK) antagonist abolished both BDNF and estradiol-mediated effects at the synapse. Although less well established, there is some evidence that progesterone may also upregulate expression and enhance the release of BDNF [12, 13], which in turn is thought to mediate some of progesterone's neuroprotective effects [14].

N-Methyl-D-aspartate-receptor (GluN) dysfunction is a common pathophysiological feature in disorders of cognitive functioning and can lead to alterations in neuronal and cortical plasticity [15]. Glutamate activity at the postsynaptic NMDA receptor is thought to critically mediate long-term potentiation (LTP) as well as spinogenesis [16, 17]. Both neurological processes are thought to underlie estradiol-dependent cognitive enhancement [18–20].

NMDA receptors are ligand-gated ion channels essential to excitatory neurotransmission. Receptors are heteromeric tetramers containing at least one NMDA-R1 (GluN1) subunit, with one or more GluN2 or GluN3 subunits [21]. There are four (A–D) glutamate-binding GluN2 subunits, and the expression of these subunits changes in a region- and development-specific manner [16]. GluN2A and GluN2B subunits have both been associated with LTP and cognitive function [22], and this is thought to be due to downstream activation of ERK [23].

Estradiol has been shown to modulate NMDA receptor-dependent activity [24] and expression within the hippocampus [25]. Furthermore, estradiol regulation of LTP has been shown to be mediated via activation of NMDA-R as the NMDA-R antagonist MK-801 was shown to abolish the effects of estradiol on LTP and spine density [26]. The involvement of progesterone and androgens in NMDA-R modulation however has received far less attention. However, one study reported a DHT-induced increase in the expression of the GluN1 subunit within the hippocampus of adult mice, suggesting beneficial effects of androgens on NMDA-R expression [27]. Postsynaptic density protein 95 (PSD-95) is a scaffolding protein found at excitatory glutamatergic synapses responsible for docking NMDA-Rs to the postsynaptic membrane, and its expression in the hippocampus has been shown to be altered by estradiol in rats [28] and testosterone in mice [29].

However, the majority of studies assessing the effects of steroid hormones on NMDA-R expression have been done in adult rodents. The first aim of this study was to investigate how alterations to sex steroid hormones during adolescent development modulate NMDA-R expression in the hippocampus. The second aim of this study was to investigate whether the effects of sex steroid hormones on NMDA-R/PSD-95 expression may be altered in BDNF heterozygous mice.

We further sought to determine whether expression patterns differed in the dorsal versus ventral hippocampus as lesion studies in mice have identified that these distinct regions specifically regulate different behaviours (e.g., dorsal hippocampus regulates spatial memory; ventral hippocampus regulates stress and emotional processing) [30]. We report highly specific effects of sex steroid hormones on NMDA-R expression that are dependent on region (dorsal versus ventral) and BDNF genotype.

## 2. Materials and Methods

### 2.1. Animals

Male and female WT and BDNF heterozygous (^+/−^) mice of C57BI/6 background were bred from an existing colony at the Florey Institute of Neuroscience and Mental Health. Mice were housed in same sex groups of 3–5. All mice were housed under standard conditions, with individually ventilated cages and ad libitum access to water and mouse chow. All experiments were approved by the Florey Animal Ethics Committee.

### 2.2. Gonadectomy and Sex Steroid Hormone Implants

Female mice were either sham-operated or ovariectomized (OVX) at prepubescence (5 weeks) and were simultaneously implanted with 1 cm of silastic tubing (1.98 mm I.D., 3.18 mm O.D.) filled with 0.5 cm 17*β*-estradiol (Sigma-Aldrich, St. Louis, MO, USA), 0.5 cm progesterone or empty implant. Please refer to Hill et al. for surgical technique details [8]. Briefly, mice were anaesthetized and received a single 5 mg/kg injection of the analgesic, carprofen (Rimadyl®, Pfizer, Sandwich, Kent, UK), and a 5 mg/kg injection of Baytril® (enrofloxacin, Bayer, Leverkusen, Germany) antibiotic to limit pain and discomfort. The ovaries were removed on both sides through an incision in the muscle tissue at the lower back. To insert the implant, a 5 mm skin incision on the nape of the neck was made and the implant was inserted into the space between the skin and the muscle. The incisions on the skin were closed with Michel clips and swabbed with the antiseptic povidone-iodine (Betadine®, Virginia, QLD, Australia) and the topical antibiotic, Tricin (zinc bacitracin, neomycin sulfate, and polymyxin B sulfate, Rutherford, NSW, Australia).

Male mice were anaesthetized in a similar manner to female mice then placed in a supine position. Briefly, a shallow 1-2 cm incision was made along the midline through the skin of the scrotum, the connective tissue was separated, and the testes were located and gently squeezed out. The fat and connective tissue were separated from the testicle and epididymis, the blood vessels and vas deferens were ligated using silk suture thread, and the testicle was removed. After both testes are removed, the scrotum is suture-closed using individual horizontal mattress stitches. The sham treatment group received a sham surgery; this was performed in the same way, with an incision made in the scrotum but the testes were left intact. Following castration, mice were turned over to a prone position and 1.5 cm silastic tubing containing testosterone (Sigma-Aldrich, St. Louis, MO, USA) or dihydrotestosterone (DHT) (Sigma-Aldrich, St. Louis, MO, USA) or empty tubing was implanted subcutaneously through a single incision at the nape of the neck.

### 2.3. Hippocampal Dissections

Male and female mice were culled at 14 weeks between the time of 1200 h and 1700 h by cervical dislocation; brains were then collected and snap-frozen at −80°C. Brains were dissected at 4°C. To extract the hippocampus, the forebrain from the level of the optic chiasm was first removed with a surgical blade, and then, the hypothalamus was dissected out from the midbrain using curved forceps. The pons and cerebellum were then pulled apart from the midbrain at the level of the corpus callosum to reveal the hippocampus which was separated from the cortex, then further dissected in half to separate the dorsal and ventral regions from the right and left hemispheres. All dissected regions were then snap-frozen on dry ice before being stored at −80 degrees for future use.

### 2.4. Protein Extraction

Frozen tissue samples were weighed, and the appropriate amount of RIPA lysis buffer (1 ml/100 *μ*g of tissue, 150 mM sodium chloride, 1.0% sodium dodecyl sulfate, 50 mM Tris pH 8.0, protease inhibitor cocktail set III (1 : 200, Merck, Kilsyth, VIC, Australia), and phosphatase inhibitor cocktail set IV (1 : 50, Merck, Kilsyth, VIC, Australia)) was added to the samples before homogenization with a hand-held homogenizer. Homogenized samples were then left on ice for 10 minutes before rotating slowly for an hour at 4°C. Samples were then centrifuged for 15 mins at 14000 ×g, at 4°C. Protein containing supernatant was carefully isolated, and a bicinchoninic acid (BCA) protein assay was performed (Thermo Scientific, Rockford, IL, USA).

### 2.5. Western Blotting

Western blotting was performed as previously described [31]. Briefly, an equal volume of loading buffer (0.4 M Tris, pH 6.8, 37.5% glycerol, 10% SDS, 1% 2-mercaptoethanol, 0.5% bromphenol blue, dH2O) was added to 50 *μ*g protein samples and denatured at 95°C for 10 minutes before SDS-polyacrylamide gel electrophoresis. Protein samples were then transferred from the polyacrylamide gel to 0.22 *μ*m nitrocellulose membrane, and Ponceau stain (Sigma-Aldrich) was utilised to confirm successful protein transfer. Membranes were then incubated in 5% milk in TBST (20 mM Tris base pH 7.5, 150 mM NaCl, 0.01% Tween 20) for 1 hour at room temperature to prevent nonspecific antibody binding. Membranes were briefly washed in TBST before primary antibody was applied. Primary antibodies were diluted in 5% bovine serum albumin (BSA) (Sigma-Aldrich) in TBST, including rabbit anti-NMDA-R (1 : 200; Abcam, ab110, GluN2B), rabbit anti-NMDA-R 2A (1 : 1000; Abcam, ab14596 GluN2A subunit), rabbit anti-PSD-95 (1 : 1000; Abcam, ab18258), rabbit anti-NMDAR1 (1 : 1000; Cell Signaling, 4204), mouse anti-*α*-tubulin (1 : 5000, Abcam, ab7291), and mouse anti-*β*-actin (1 : 10,000; Sigma, a5316). Membranes were incubated overnight with primary antibody at 4°C on a shaker. In the following day, membranes were washed twice for 15 mins in TBST on a shaker before a 1.5-hour incubation at room temperature with either anti-mouse or anti-rabbit IgG HRP-linked secondary antibodies (1 : 2000; Cell Signaling Technology; Danvers, MA, USA). After a further 3 × 15 min wash in TBST, membranes were then exposed to LumiGlo Chemiluminescence (Cell Signaling Technology; Danvers, MA, USA) for 1 minute and imaged using the Luminescence Image analyser (LAS-4000; FujiFilm Life Science, Stamford, CT, USA). The images were analysed using TotalLab or CLIQS (Newcastle, UK) software. All targets were normalised to either *β*-actin or *α*-tubulin loading control. Expression levels were then represented as relative to the average of WT sham controls. For each target, at least two Western blot experiments were performed and individual values were averaged.

### 2.6. Statistics

For each protein target, two separate ANOVAs were performed, one analysing intact versus gonadectomised (OVX or CAST) mice and a second analysis for gonadectomised versus gonadectomised + treatment (estradiol, progesterone, testosterone, and DHT). Separate ANOVAs were required as we deemed it biologically incorrect to compare gonadectomised + treatment groups to intact controls. If an OVX × genotype interaction was found, Sidak's multiple comparisons post hoc test was performed. If an overall effect of treatment was found, Tukey's post hoc analysis was performed to further investigate individual treatment effects (i.e., estradiol or progesterone). If a genotype × treatment interaction was found, Tukey's post hoc analysis was performed to further investigate this interaction. Significance was set at *p* < 0.05. A value of *p* < 0.1 was considered a trend.

## 3. Results

### 3.1. GluN1 Expression in Female Hippocampus

**Figure 1:**
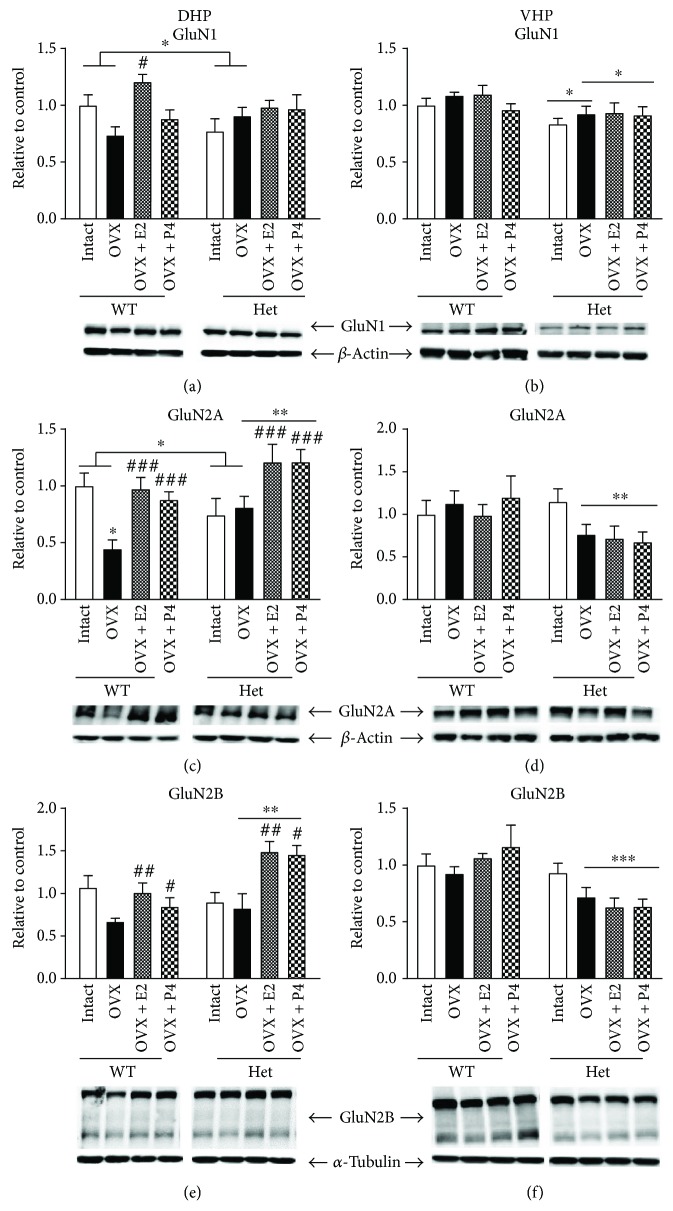
Protein expression of NMDA receptors in the dorsal (DHP) and ventral (VHP) hippocampus of female sham ovariectomized + placebo (intact), ovariectomized + placebo (OVX), OVX + estradiol (E2), and OVX + progesterone- (P4-) treated mice. (a) GluN1 expression in the DHP, (b) GluN1 expression in the VHP, (c) GluN2A expression in the DHP, (d) GluN2A expression in the VHP, (e) GluN2B expression in the DHP, and (f) GluN2B expression in the VHP. *N* = 5–7 per treatment group. ^∗^*p* < 0.05, ^∗∗^*p* < 0.001, and ^∗∗∗^*p* < 0.0001. Error bars represent the standard error of the mean (SEM). Two-way ANOVA was performed. Bars with connecting horizontal lines show significant interactions (OVX × genotype), flat bars represent significant main effects of the genotype, and ∗ above the OVX bar represents post hoc significant differences between OVX and sham control, while # above hormone-treated bars represents significant differences between hormone-treated OVX groups and OVX placebo. ^#^*p* < 0.05, ^##^*p* < 0.01, and ^###^*p* < 0.005.

Two-way ANOVA analysing intact versus OVX groups showed a significant OVX and genotype interaction (*F* (1, 20) = 4.999, *p* = 0.03; Figure 1(a)) but no main effects of genotype or OVX. While further post hoc analysis showed no significant differences, this significant interaction results from reduced GluN1 expression in WT OVX, but slightly elevated levels in the BDNF hets. Further two-way ANOVA analysing OVX and OVX + treatment groups revealed a significant effect of the treatment (*F* (2, 27) = 4.65, *p* = 0.01) and a trend for a treatment × genotype interaction (*F* (2, 27) = 2.571, *p* = 0.09; Figure 1(a)). Post hoc analysis for the main effect of treatment here showed a significant increase in GluN1 expression following estradiol (*p* = 0.018) but not progesterone treatment. Post hoc analysis for the genotype × treatment interaction showed that this increase in GluN1 expression was limited to WT mice with a significant difference between WT OVX and WT OVX + E2 (*p* = 0.014), but no significant difference between het OVX and het OVX + E2 (Figure 1(a)). In the VHP, a significant main effect of genotype was found when analysing intact and OVX groups (*F* (1, 23) = 4.593, *p* = 0.04); however, no effect of OVX and no genotype × OVX interaction was found (Figure 1(b)). In addition, when analysing OVX and OVX + treatment groups, a significant effect of genotype was found (*F* (1, 33) = 7.135, *p* = 0.01; Figure 1(b)) with BDNF het mice showing reduced expression. No significant effect of treatment and no genotype × treatment interaction were found here (Figure 1(b)).

### 3.2. GluN2A Expression in Female Hippocampus

In the female DHP, a significant effect of OVX (*F* (1, 21) = 4.705, *p* = 0.04) and a significant OVX × genotype interaction (*F* (1, 21) = 7.603, *p* = 0.01) were found for GluN2A expression (Figure 1(c)). Here, Sidak's multiple comparisons test showed a significant reduction in GluN2A expression following OVX in WT (*p* = 0.0038) but not BDNF het mice. Significant effects of treatment (*F* (2, 31) = 9.937, *p* = 0.0005) and genotype (*F* (1, 31) = 9.789, *p* = 0.0038) were also found for GluN2A expression, with both BDNF het genotype and treatments appearing to increase GluN2A expression, while no genotype × treatment interaction was found. Here, Tukey's post hoc comparison for the main effect of treatment showed that both estradiol (*p* = 0.0007) and progesterone (*p* = 0.006) increased GluN2A expression (Figure 1(c)). In the VHP, no effect of genotype or OVX was found; however, a trend for an OVX × genotype interaction was found (*F* (3, 46) = 1.737, *p* = 0.09; Figure 1(d)). In addition, when analysing OVX and OVX + treatment groups, we found a significant effect of genotype on GluN2A expression (*F* (1, 34) = 9.492, *p* = 0.0041) with BDNF het mice again showing reduced expression. No effect of treatment and no genotype × treatment interaction were found for GluN2A expression in the VHP of females (Figure 1(d)).

### 3.3. GluN2B Expression in Female Hippocampus

In the female DHP, we found a trend for an effect of OVX (*F* (1, 21) = 3.574, *p* = 0.07) but no effect of genotype and no genotype × OVX interaction. A significant effect of treatment (*F* (2, 30) = 6.905, *p* = 0.0034) and a significant effect of genotype (*F* (1, 30) = 11.91, *p* = 0.0017) were found for GluN2B expression; however, no significant genotype × treatment interaction was found. Here, once again, it appeared that both treatments and BDNF genotype increased GluN2B expression. Tukey's post hoc analysis for the main effect of treatment revealed a significant increase in GluN2B expression following both estradiol (*p* = 0.0067) and progesterone (*p* = 0.0272) (Figure 1(e)). In the female VHP, a trend for an effect of OVX (*F* (1, 24) = 2.978, *p* = 0.09) was found, but no significant effect of genotype and no OVX × genotype interaction were found. However, when analysing OVX and OVX + treatment groups, a significant effect of genotype (*F* (1, 35) = 26.46, *p* < 0.0001) was found for GluN2B expression, with BDNF het mice again showing reduced expression compared to their WT treatment-matched controls (Figure 1(f)). No effect of treatment and no treatment × genotype interaction were found.

### 3.4. TrkB, pTrkB, and pERK Expression in the Female DHP

**Figure 2:**
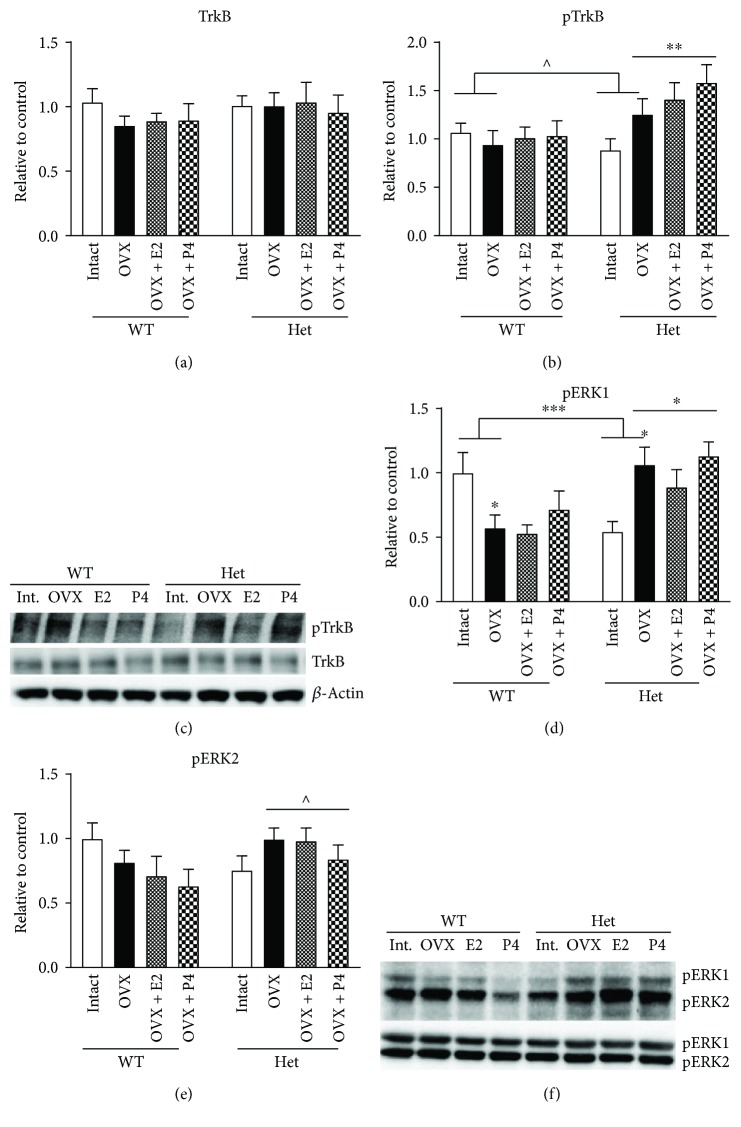
Protein expression of TrkB, pTrkB, and pERK1/2 in the dorsal (DHP) hippocampus of female sham ovariectomized + placebo (intact), ovariectomized + placebo (OVX), OVX + estradiol (E2), and OVX + progesterone- (P4-) treated mice. (a) Full-length TrkB in the DHP, (b) phosphorylated TrkB (Tyr515) in the DHP, (c) representative blots of TrkB and pTrkB, (d) phosphorylated ERK1 in the DHP, (e) phosphorylated ERK2 in the DHP, and (f) representative blots of pERK1 and 2 and total ERK1 and 2. *N* = 5–7 per treatment group. ^^^*p* < 0.1, ^∗^*p* < 0.05, ^∗∗^*p* < 0.001, and ^∗∗∗^*p* < 0.0001. Error bars represent the standard error of the mean (SEM). Two-way ANOVA was performed. Bars with connecting horizontal lines show significant interactions (OVX × genotype), flat bars represent significant main effects of genotype, and ∗ above the OVX bar represents post hoc significant differences between OVX and sham control.

In the dorsal hippocampus of female mice, we noted that the BDNF hets tended to show a hypersensitive response to hormone treatment in terms of GluR2A and 2B expression. To investigate this further, we analysed upstream regulator of GluR2 expression, TrkB, as well as a common downstream intracellular molecule, ERK. Here, no effect of OVX, treatment, or genotype was found for full-length TrkB expression (Figures 2(a) and 2(c)). However, when analysing TrkB phosphorylation at tyrosine 515 in control and OVX groups, we found a trend for an interaction with levels remaining stable in controls but elevated in BDNF het mice (Figures 2(b), 2(c), and 2(f); (1, 24) = 3.499, *p* = 0.07). Furthermore, when analysing OVX and OVX + treatment groups, there was an overall effect of genotype (*F* (1, 35) = 10.96, *p* = 0.0022) with BDNF hets showing elevated pTrkB expression. For phosphorylated ERK1, we found a significant interaction when comparing control and OVX groups (Figures 2(d) and 2(f); *F* (1, 22) = 14.86, *p* = 0.0009) with OVX causing a reduction in pERK1 in WT mice (*p* = 0.04) but an increase in BDNF het mice (*p* = 0.01). When analysing OVX and OVX + treatment groups, we once again found the main effect of genotype (Figures 2(d) and 2(f); *F* (1, 36) = 18.99, *p* = 0.0001), with BDNF het mice showing elevated pERK1 expression, similar to pTrkB. For pERK2, no significant effects of OVX or genotype were found when comparing controls and OVX; however, when comparing OVX and OVX + treatment groups, there was a trend for an effect of genotype (Figures 2(e) and 2(f); *F* (1, 34) = 3.835, *p* = 0.058), once again with BDNF hets showing elevated expression.

### 3.5. GluN1 Expression in Male Hippocampus

**Figure 3:**
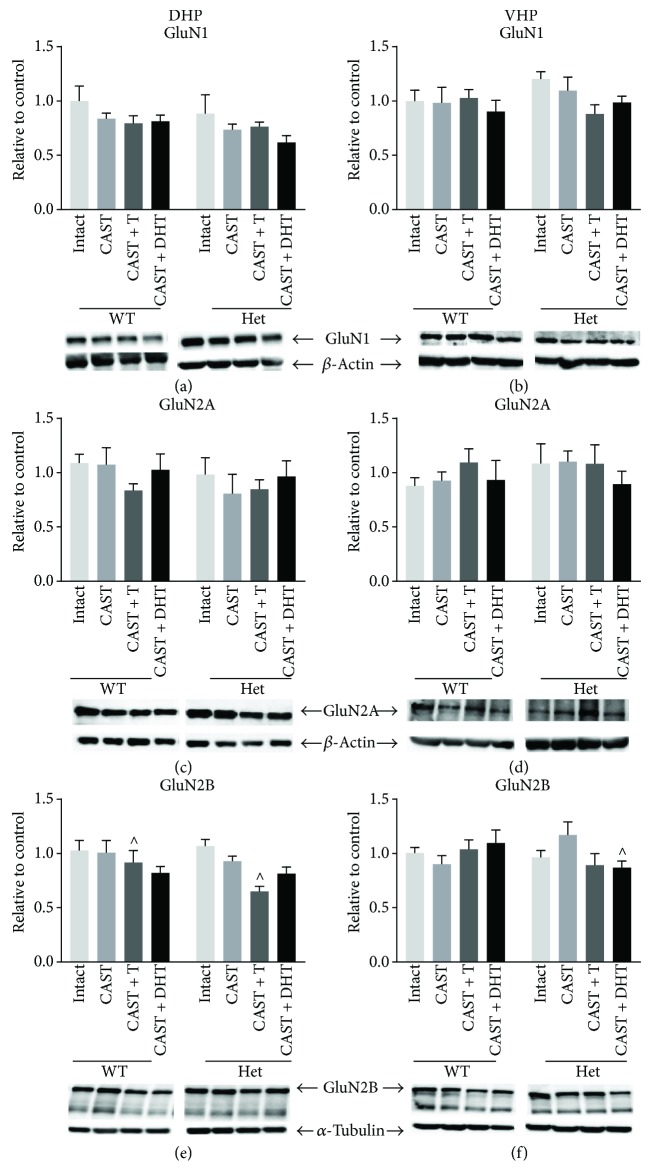
Protein expression of NMDA receptors in the dorsal (DHP) and ventral (VHP) hippocampus of male sham castrated + placebo (intact), castrated + placebo (CAST), CAST + testosterone (T), and CAST + dihydrotestosterone- (DHT-) treated mice. (a) GluN1 expression in the DHP, (b) GluN1 expression in the VHP, (c) GluN2A expression in the DHP, (d) GluN2A expression in the VHP, (e) GluN2B expression in the DHP, (f) GluN2B expression in the VHP. Representative blots for each NMDA receptor subtype as well as the loading control. *N* = 5–7 per treatment group. ^^^*p* < 0.1. Error bars represent the standard error of the mean (SEM). Two-way ANOVA was performed. ^ above the CAST + hormone-treated bars represents post hoc trend showing a difference between CAST + hormone-treated groups and CAST placebo.

In both the dorsal and ventral hippocampus, there was no effect of genotype, castration, and treatment and no interactions on GluN1 expression (Figures 3(a) and 3(b)).

### 3.6. GluN2A Expression in Male Hippocampus

In both DHP and VHP, no effect of genotype, castration, and treatment and no interactions were found on GluN2A expression (Figures 3(c) and 3(d)).

### 3.7. GluN2B Expression in Male Hippocampus

In the DHP, there was no effect of genotype or castration on GluN2B expression when analysing intact and CAST groups. However, a trend for an effect of genotype (*F* (1, 30) = 3.397, *p* = 0.075) and a trend for an effect of treatment (*F* (2, 30) = 3.18, *p* = 0.056) were found when analysing CAST and CAST + treatment groups. Post hoc comparisons for the main effect of treatment showed a trend for a reduction in GluN2B in CAST + testosterone compared to CAST mice (*p* = 0.06) (Figure 3(e)). While this reduction following testosterone treatment seemed to be more pronounced in BDNF het mice, no significant genotype × treatment interaction was found here. However, in the VHP, a significant genotype × treatment interaction was found (*F* (2, 28) = 3.606, *p* = 0.04; Figure 3(f)). Here, there was a trend for a reduction in GluN2B following DHT treatment in BDNF het mice (*p* = 0.08), while in WT mice, there was no significant effect of DHT.

### 3.8. PSD-95 in Female Hippocampus

**Figure 4:**
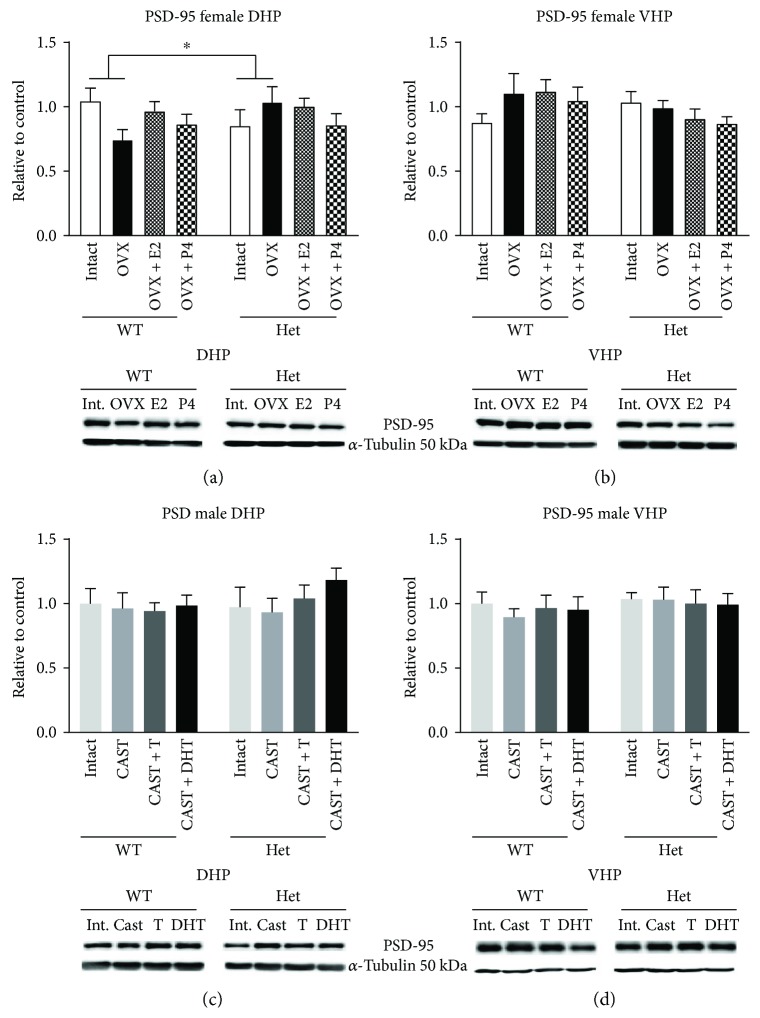
Protein expression of PSD-95 in the dorsal (a) and ventral (b) hippocampus of female sham ovariectomized + placebo (intact), ovariectomized + placebo (OVX), OVX + estradiol (E2), and OVX + progesterone- (P4-) treated mice. Protein expression of PSD-95 in the dorsal (c) and ventral (d) hippocampus of male sham castrated + placebo (intact), castrated + placebo (CAST), CAST + testosterone (T), and CAST + dihydrotestosterone- (DHT-) treated mice. *N* = 5–7 per treatment group. ^∗^*p* < 0.05. Error bars represent the standard error of the mean (SEM). Bars with connecting horizontal lines show significant interactions (OVX × genotype).

A significant genotype × OVX interaction (*F* (1, 24) = 5.200, *p* = 0.0318) was found for PSD-95 expression in the DHP of female mice. Here, while post hoc comparisons showed no significant effects, it appears that WT mice show a reduction in PSD-95 following OVX, while in BDNF het mice, expression levels appear greater following OVX (Figure 4(a)). No overall effects of genotype or OVX were found. No significant effects of genotype or treatment were found when analysing OVX and OVX + treatment groups.

In the female VHP, no significant effects of genotype, OVX, treatment, or interactions were found (Figure 4(b)).

### 3.9. PSD-95 in Male Hippocampus

In the male DHP and VHP, no significant effects of genotype, castration, treatment, or genotype × treatment interactions were found for PSD-95 expression (Figures 4(c) and 4(d)).

## 4. Discussion

We report striking region-specific effects of sex steroid hormones and BDNF genotype on hippocampal NMDA-R and PSD-95 expression. In female mice, sex steroid hormone manipulation significantly altered GluN1, GluN2A, and GluN2B expression only in the DHP, with no effects of OVX or treatment found within the VHP. This aligns with previous reports in rats which show a beneficial effect of estradiol on adult hippocampal GluN1 protein expression [32], suggesting that adolescent hormonal regulation of GluN1 is similar to adulthood. A significant genotype × OVX interaction was found for GluN1 expression in the female DHP. Here, OVX reduced expression levels and estradiol recovered expression in the WT DHP, but no such effects were apparent in the BDNF het mice, suggesting that this effect of estradiol on GluN1 is BDNF dependent.

Similar genotype × OVX effects were found for GluN2A, GluN2B, and the NMDA-R scaffolding protein, PSD-95. However, hormone-treated BDNF het mice show highly elevated expression levels of GluN2A and GluN2B well above WT treatment-matched controls. Physiological response to estrogenic compounds is thought to operate on a U-shaped dose-response curve [33]. The fact that WT mice do not upregulate levels of NMDA subunits beyond that found in intact mice shows an appropriate response to hormone manipulation. In contrast, the hypersensitive response of BDNF het mice treated with estradiol and progesterone suggests a decoupling from the normal U-shaped response curve. This “hypersensitive” response of BDNF het mice to hormone manipulation is unlikely to be due to alterations in estrogen receptor expression as we have previously shown no changes in either ER*α* or ER*β* expression in these mice [9]. This is in contrast to previous reports in rats whereby OVX in adulthood caused a long-lasting reduction in hippocampal ER*α* expression [34] and highlights the differential effect of OVX during adolescence compared to adulthood. However, another upstream regulator of GluN2 expression that may modulate this “hypersensitive” response is the TrkB receptor [35]. Therefore, we analysed the expression of full length as well as phosphorylated TrkB (tyr515) and indeed found a significant effect of genotype in both OVX and OVX + hormone-treated mice. Adding strength to the above data, when analysing downstream intracellular ERK phosphorylation, a similar pattern of expression was observed. Hence, we suggest that the aforementioned resilience of BDNF het mice to a loss of circulating sex hormones may be due to the upregulation of pTrkB, which in combination with high levels of either estradiol or progesterone causes further upregulation of GluN2 subunits. Further experiments using TrkB inhibitors would be required to confirm this suggestion.

While we anticipated beneficial effects of estradiol on GluN expression in WT mice, the beneficial effects of progesterone were in opposition to previous reports which found that progesterone and estradiol + progesterone treatment decrease NMDA-specific binding within the frontal cortex of rats [24]. Here, species and region differences may account for these discrepancies. Furthermore, this may reflect the vulnerability of the adolescent period when manipulating sex steroid hormone levels.

In contrast to the DHP, sex steroid hormone manipulation had no effect on NMDA-R expression in the VHP. Relatively few reports examine region specificity with regard to estrogenic signalling within the hippocampus. However, one study did find that estradiol had the ability to alter dorsal but not ventral hippocampal seizures [36], demonstrating estradiol's propensity to affect the DHP. Within the VHP, female BDNF het mice expressed significantly lower levels of GluN1, GluN2A, and GluN2B subunits compared to WT mice, and in regard to the GluN2 subunits, this was specifically within the OVX and OVX + treatment groups with intact BDNF het mice showing similar levels to their WT treatment-matched controls. Therefore, it appears that the combined effect of OVX and genotype reduced GluN2 expression and neither estradiol nor progesterone could recover this. This effect of genotype in OVX and OVX + treatment mice may be due to stress associated with the OVX surgery—to which BDNF het mice may be more vulnerable. However, intact controls did undergo sham procedures which consisted of exactly the same surgery but without the removal of the ovaries. Alternatively, it may be that in BDNF het mice both estradiol and progesterone are required in conjunction to maintain NMDA-R expression levels within the VHP.

Previous behavioural data published by our laboratory on the same cohort of female mice found preadolescent OVX in WT mice to cause Y-maze deficits, which were relieved by estradiol but not progesterone treatment, albeit the progesterone group was trending toward an improvement in Y-maze behaviour [9]. Y-maze is a dorsal hippocampus-dependent task; therefore, this reduction in GluN2A and GluN2B subunits observed provides a possible mechanism through which OVX and subsequent estradiol and progesterone treatment affect spatial cognition in WT mice. No significant reduction in GluN2A/ GluN2B subunit expression was observed in BDNF het mice following OVX. Interestingly, OVX in BDNF het mice did not create deficits in the Y-maze task but actually seemed to improve performance [9]. Along with this line, we would expect BDNF het mice treated with estradiol and progesterone to have shown enhanced performance on the Y-maze as levels of GluN2A and GluN2B are significantly elevated in these treatment groups. However, no such further enhancement is observed [9]. Reports suggest that NMDA-R activation promotes activity-dependent release of BDNF from the presynaptic membrane. Park and colleagues [37] showed that induction of LTP is dependent on both NMDA-R action and BDNF release. Despite increased levels of GluN2A and GluN2B found in hormone-treated BDNF hets, no increase in cognitive performance was observed. Perhaps the reduction in BDNF in these animals prevents LTP induction from occurring, thus no observable increase in cognitive function. Indeed, in our previous study, we found that levels of BDNF corresponded with Y-maze performance in WT but not BDNF het mice [9]. Alternatively, it may be that overexpression of GluN2A and GluN2B subunits in BDNF het mice, particularly within the estradiol treatment group, is causing excitotoxicity, leading to impaired Y-maze performance [9]. Indeed, neuroprotective effects of estradiol against NMDA toxicity are thought to be mediated by BDNF [38]; therefore, in the absence of BDNF (BDNF heterozygous), perhaps this protection is lost. Further studies to identify whether the changes we see in GluN expression are synaptic or extrasynaptic may help to clarify this effect as previous studies suggest that the activation of synaptic GluN leads to synaptic plasticity and cell survival, while extrasynaptic activation can lead to excitotoxic cell death [39]. Indeed, a previous *in vitro* report showed that low concentration of estradiol (1 nM) increased the membrane expression of cortical GluN1, GluN2B, and PSD-95, while a higher concentration had no effect but reduced GluN2A [40]. Further in vivo studies assessing varying concentration of estradiol treatment would certainly be of interest here to fully elucidate the beneficial potential of estradiol on hippocampal synaptic plasticity.

In the male mice, we found no significant effects of castration, hormone treatment, or genotype on GluN1 expression. This is in contrast with previous studies reporting detrimental effects of androgen receptor deletion on GluN activation and temporal processing [41] and enhancement of hippocampal dendritic spine density and GluN1 expression following DHT administration [27]. These discrepancies may relate to the timing of our chronic treatments during the adolescent period as opposed to adulthood.

In contrast, a reduction in GluN2B expression was observed following testosterone treatment in the DHP of both WT and BDNF het male mice. Consistency between WT and BDNF het mice in GluN2B expression suggests this process is independent of BDNF. A divergent pattern of expression of GluN2B is observed in the VHP, whereby DHT but not testosterone treatment reduces expression of GluN2B exclusively in BDNF het mice. This finding suggests that reduced BDNF in the VHP makes the region more susceptible to androgen-specific signalling.

Interestingly, changes in GluN subunit expression in response to gonadal hormone manipulation are found predominantly in the DHP. This suggests some commonality between males and females, in that sex hormone manipulation preferentially affects the DHP. More robust effects found in female compared to male mice are in line with the current literature surrounding estradiol's dominant role in synaptic plasticity [42]. Male animals may be less responsive to changes in circulating sex hormones due to the relatively higher levels of hippocampal estradiol found in the male compared to female. Using mass-spectrometric analysis, Ooishi and colleagues [43, 44] detected concentrations of estradiol in the hippocampus of male and female rats at 8 nm and 0.5–3 nm, respectively. Interestingly, Hojo et al. reports only a marginal decrease in estradiol levels within the hippocampus of male rats following castration [43]. Although this report is in rats, this does provide a possible explanation for relative GluN stability following castration in male mice.

In conclusion, we report significant effects of sex steroid hormones during adolescence on GluN1, 2A, 2B, and PSD-95 expression that are mainly restricted to the dorsal hippocampus, suggesting region-specific effects. Furthermore, we found significant genotype × OVX and genotype × treatment interactions within the female DHP, whereby BDNF het mice tended to respond in an opposite manner to WT mice following OVX and hormone treatments tended to increase GluN2 expression to superphysiological levels. This hypersensitive response of BDNF het mice may be due to altered pTrkB signalling. Therefore, in periods of significant change in sex hormone levels, such as puberty, pregnancy, or menopausal, whereby cognitive ability tends to be affected, BDNF may be a mediator of these effects and thus may represent a novel therapeutic target for postmenopausal-associated cognitive decline, including dementia [45].
